# Antidiarrheal and Antisecretory Effect of 80% Hydromethanolic Leaf Extract of *Moringa stenopetala* Baker f. in Mice

**DOI:** 10.1155/2022/5768805

**Published:** 2022-01-30

**Authors:** Menbere Getaneh Woldeyohannes, Gelila Tamyalew Eshete, Alfoalem Araba Abiye, Abebe Ejigu Hailu, Solomon Assefa Huluka, Wondmagegn Tamiru Tadesse

**Affiliations:** ^1^Department of Pharmacology and Clinical Pharmacy, School of Pharmacy, College of Health Sciences, Addis Ababa University, Addis Ababa, Ethiopia; ^2^Department of Pharmaceutics and Social Pharmacy, School of Pharmacy, College of Health Sciences, Addis Ababa University, Addis Ababa, Ethiopia

## Abstract

**Introduction:**

In Ethiopia, different medicinal plants have been claimed and used to treat diarrheal diseases. However, these claimed effects for most medicinal plants have not been scientifically verified. One of such plants in Ethiopian folkloric medicine is *Moringa stenopetala,* which is usually consumed as a vegetable in southern Ethiopia. Thus, this study aimed to evaluate the antidiarrheal and antisecretory effects of 80% methanolic leaf extract of *Moringa stenopetala* in different mice models.

**Method:**

Using Swiss albino mice, castor oil-induced diarrhea, charcoal meal-based gastrointestinal motility, and castor oil-induced secretion models were employed to assess antidiarrheal activity. In all of the test models, animals were randomly assigned into five groups consisting of six animals in each group. Group I received 0.5 ml of the vehicle (2% tween-80), while group II was treated with standard drug (3 mg/kg loperamide) in the respective models, whereas groups III to V received 150, 300, and 450 mg/kg of the methanolic leaf extracts of *Moringa stenopetala*. Onset, frequency, consistency, and weight of stool (diarrhea) were recorded, and different parameters and percentage proportions were calculated. Data were analyzed using one-way ANOVA followed by Tukey's test, and *p* < 0.05 was considered statistically significant at 95% confidence of interval.

**Result:**

In the castor oil-induced diarrheal model, the percentage inhibition of diarrhea was 48.5, 58.6, and 60% for the respective doses of 150, 300, and 450 mg/kg of the extract. And, the extract showed a 36.8, 54.4, and 55.9% reduction of peristalsis in charcoal meal-based gastrointestinal motility test compared to the negative control group. Moreover, in the antisecretory assay, the 150, 300, and 450 mg/kg doses of MEMS inhibited fluid contents of the stool by 11.5, 54.54, and 61.82%, respectively, relative to the vehicle-treated group.

**Conclusion:**

The findings revealed that the 80% methanolic leaf extract of *Moringa stenopetala* extract has shown antidiarrheal activity.

## 1. Introduction

Diarrhea is the passage of loose, unformed, or liquid stools at least three times a day. Diarrhea is the fifth cause of death next to preterm birth complications, acute respiratory infections, intrapartum-related complications, and congenital anomalies among under-five children, accounting for 15% of all deaths of children globally. And it is responsible for killing about 525,000 children each year [1]. The burden of the disease is higher in developing countries where access to health care, safe water supply, and hygienic conditions are inadequate and poor [2].

Although the different classes of medications are currently used to manage diarrhea, most of them are associated with several adverse effects [3]. For instance, abdominal discomfort, constipation, nausea, vomiting, bronchospasm, and dry mouth are reported from the use of the different contemporary antidiarrheal agents [4]. Moreover, the use of antimicrobials is also associated with the incidence of drug resistance, superinfection, and other effects [5]. The use of oral rehydration therapy is very important to manage diarrheal conditions, but it is not effective in alleviating the frequency of loose stool and high output states [3].

Despite the advancement of modern medicine, the World Health Organization (WHO) reported that about 80% of the world's population still relies on traditional medicine for basic health needs because of its affordability and accessibility. WHO also encourages the scientific validation of traditional medicine as a source of effective and affordable treatments [6, 7]. Different medicinal plants have been used for various ailments, including diarrheal conditions. It is believed that drugs sourced from medicinal plants are safe and with minimal adverse events [8]. Herbal medicine can be a source of better and effective treatment alternatives only if research activities focus on traditional medicine. Therefore, screening and evaluating medicinal plants with antidiarrheal potential can contribute to the development of new candidate drugs [9].

In Ethiopia, like many developing countries, medicinal plants provide a vital contribution to human health care [9, 10]. Several plants have been widely used to manage diarrhea by traditional medical practitioners [11]. *Moringa stenopetala* is one of such medicinal plants considered in the management of diarrhea. Due to its economic, nutritional, and medicinal values, this plant is widely cultivated in Ethiopia, especially in the southern part [12]. The literature outlines that *M. stenopetala* is used in a range of conditions, including leishmaniasis, malaria, trypanosomal infection, diabetes mellitus, and hypertension management [13]. Moreover, the leaf of *M. stenopetala* is reported to have an antimicrobial effect [14]. Local communities use *M. stenopetala* for unspecified stomach problems such as abdominal cramps, diarrhea, and diarrhea-associated conditions [15, 16]. In southern Ethiopia, *M. stenopetala* is also used to treat indigestion and a form of diarrhea like dysentery [13, 17]. From the same family, *Moringa oleifera* demonstrated significant protection against experimentally induced diarrhea [18, 19]. However, no data have been found regarding the antidiarrheal activity of *M. stenopetala* so far. Therefore, this study aimed to investigate the antidiarrheal and antisecretory activities of 80% methanolic leaf extract of *M. stenopetala* (MEMS) in mice models.

## 2. Materials and Methods

### 2.1. Drugs and Chemicals

The following drugs and chemicals were used in this experiment: methanol (RANKEM, India), tween-80 (Sigma-Aldrich, UK), activated charcoal (Lab. Reagent, India), atropine sulfate (AdvaCare, USA), castor oil, and loperamide were obtained from a local retail outlet in Addis Ababa.

### 2.2. Collection, Preparation, and Extraction of Plant Material

Fresh leaves of *M. stenopetala* were collected from the local market from Lante (near Arba Minch), Gamo Zone, Ethiopia, and authenticated by a taxonomist at the National Herbarium, College of Natural Sciences, Addis Ababa University (AAU), and a sample was kept there (voucher sample number GT/01).

The leaves were cleaned and shed-dried at room temperature and pulverized using mortar and pestle. About 200 g of the pulverized leaves was macerated with 80% methanol in a 1 : 5 ratio (100 g powder in 500 ml of 80% methanol) for 72 h and successively macerated and filtered three times using Whatman filter paper number 1 (Whatman^®^, England) by a vacuum pump. Then, the solvent was evaporated by using a rotary evaporator (Buchi Rotavapor, Switzerland) at 40°C. Then, the concentrated extract was lyophilized at −50°C under reduced pressure to get a solid product. Then, the product was packed in a tightly closed container and kept in a refrigerator until further use.

The percentage yield was calculated using the following formula:

(1) $$\begin{array}{c} \%\text{ yield of extract }=\frac{\text{weight of extract}}{\text{the initial weight of leaves macerated}}\times 100\%. \end{array}$$

### 2.3. Experimental Animals

Healthy Swiss albino mice of either sex, aged 8–10 weeks and weighing 20–30 g, were obtained from the Department of Pharmacology and Clinical Pharmacy Lab animal house, Addis Ababa University. The animals were kept in polypropylene cages (6–10 animals per cage), maintained under standard conditions (22 ± 2°C, 54–56% relative humidity, and 12 h light and 12 h dark cycle). They were provided with standard pellets and water *ad libitum*. After randomized grouping, the animals were acclimatized to the laboratory conditions for a week before the actual experiment. They were handled according to international laboratory animal use and care guidelines throughout the experiment [20].

### 2.4. Grouping and Dosing

The animals were randomly assigned into five groups, with each group consisting of six animals for each model. Group I was given a vehicle (0.5 ml of 2% tween-80) and served as a negative control. Group II was given standard drug, loperamide 3 mg/kg, and served as standard treatment control. The remaining three groups (groups III–V) received the methanol extract of MEMS at doses of 150, 300, and 450 mg/kg, respectively. The treatment doses of the extract were selected based on a prior pilot study since the 100 mg/kg dose, which was based on the acute toxicity results of the previous study, did not show an antidiarrheal effect. Based on the pilot tests, 150 mg/kg was considered as the lower dose replacing 100 mg/kg dose. The two- and threefold of the lower doses, that is, 300 and 450 mg/kg, respectively, were selected as middle and higher doses for the experimental models.

### 2.5. Castor Oil-Induced Diarrhea

Animals were screened for castor oil sensitivity just 3–4 days before the experiment. And thirty castor-oil sensitive animals were selected from the screening test and grouped into five groups, randomly, each group consisting of six animals. After 4 h of fasting, each animal was treated with 0.5 ml of castor oil perorally to induce diarrhea [21]. Thirty minutes later, animals received respective treatments as stated in the “Grouping and Dosing” section.

Then, individual animals were placed in transparent metabolic cages, the floor of which was lined with blotting paper. And each hour, the blotting paper was changed until the 4th hour. In each hour, the onset of defecation, stool consistency, frequency, and weight of the blotting paper was recorded for each animal in the respective groups during the observation period. Stool consistency was recorded based on a numerical stool score in which normal stool was assigned as 1, semisolid stool as 2, and watery stool as 3 [8].

Then, each blotting paper was kept for 48 h and air-dried. After that, the dry stool weight and stool water content were calculated by considering the weight of blotting paper without a stool, with wet stool, and with dried stool [22].

The percentage inhibition of diarrhea was calculated as follows:

(2) $$\begin{array}{c} \%\text{ inhibition of diarrhea}=\frac{\text{mean number of wet feces frequency }\left(C-T\right)}{\text{mean number of wet feces in }C}\times \;100,\\ \%\text{ weight of total fecal output}=\frac{\text{mean fecal weight of }\left(C-T\right)}{\text{mean fecal weight of }C}\times \;100,\\ \%\text{ delay of onset of diarrhea}=\frac{\text{mean onset time of defecation }\left(C-T\right)}{\text{mean onset time of defecation in }T}\times \;100, \end{array}$$

where WFC denotes the wet feces in the control group, WFT denotes the wet feces in the test group, *C* denotes the control group, and *T* denotes the test group.

### 2.6. Gastrointestinal Motility by Charcoal Meal

Animals were grouped into five groups of six animals in each group. The respective groups of animals were given 1 ml of charcoal meal orally. Thirty minutes later, all animals were treated with respective treatments as stated in the “Grouping and Dosing” section. Then, 30 minutes later, all animals were sacrificed, and the translocation of charcoal from the pylorus to the cecum was recorded in centimeters [22]. The translocation of charcoal along the length of the small intestine was calculated in terms of percent travel. The percentage inhibition of gastrointestinal motility was calculated by the following equation:

(3) $$\begin{array}{c} \%\text{ inhibition of GI motility}=\frac{\text{Mean charcoal peristalsis distance }\left(C-T\right)}{\text{Mean charcoal peristalsis distance in }C}\times \;100, \end{array}$$

where *C* denotes the control group and *T* denotes the test group.

*In vivo* antidiarrheal index was determined by using the following formula:

(4) $$\begin{array}{c} \text{ADI}=\sqrt[{3}]{D_{\text{DT}}\;\times \;G_{\text{MT}}\;\times \;N_{\text{FS}}}\text{ ,} \end{array}$$

where ADI denotes the antidiarrheal index, *D*_DT_ = % delay in defecation, *G*_MT_ = % gastrointestinal motility marked by charcoal, and *N*_FS_ = % reduction in the number of stools.

### 2.7. Antisecretory Assay

Prescreened animals were fasted for 4 h and then treated with 0.5 ml of castor oil to induce GI secretions [21]. After 30 minutes, the animals were treated with vehicle, loperamide 3 mg/kg, and extract doses at 150, 300, and 450 mg/kg, according to their respective groups. 1 h after the treatment, each animal was sacrificed, gastrointestinal fluid was milked out, and the volume of intestinal secretion was measured using a graduated measuring cylinder. Parallel to this, the weight of the intestine for each animal was recorded just before and after milking out the fluid.

### 2.8. Statistical Analysis

Data were entered, and analysis was performed using statistical package for social sciences (SPSS) version 20 (IBM^®^ SPSS^®^ Statistics). Results were expressed as mean ± standard error of the mean. One-way Analysis of variance (ANOVA) was used to determine the level of statistical significance, followed by Turkey's post hoc test. Results were considered statistically significant when the *p*-value is < 0.05 at a 95% confidence interval.

## 3. Results

### 3.1. Extraction

The percentage yield of methanolic extract of the dried leaves of *M. stenopetala* was 20% (w/w). The extract was dark-black, thick, and sticky at room temperature and readily solidified at refrigeration.

### 3.2. Effect of 80% Methanol Extract of *M. stenopetala* in the Castor Oil-Induced Diarrhea Model

MEMS and loperamide treated groups demonstrated a significant reduction in the frequency of defecation in all of the tested doses of MEMS compared to the vehicle-treated group (Table 1). The extract doses at 300 and 450 mg/kg significantly changed the stool consistency (*p* < 0.05) (Table 1). MEMS treatment significantly prolonged the onset time of diarrhea compared to the control group. Percentage inhibition of the frequency of defecations was 48.5, 58.6, and 60.6% for 150, 300, and 450 mg/kg doses of the extract, respectively. Additionally, the 150, 300, and 450 mg/kg doses of MEMS delayed the onset of diarrhea by 22.7, 56.8, and 55.1%, respectively, relative to the vehicle-treated controls.

### 3.3. Gastrointestinal Motility Test by Charcoal Meal

As shown in Table 2, MEMS doses of 150, 300, and 450 mg/kg showed a 39.32, 28.93, and 27.36% inhibition of peristalsis, respectively, relative to the vehicle-treated control group, whereas the ratio of the mean charcoal peristalsis to the mean total length of the intestine of individual groups was 0.69, 0.71, and 0.74 for the 150, 300, and 450 mg/kg of MEMS.

### 3.4. Antisecretory and Antienteropooling Assay by the Castor Oil-Induced Model

As stated in Table 3, the 150, 300, and 450 mg/kg doses of MEMS inhibited fluid contents of the stool by 11.5, 54.54, and 61.82%, respectively, relative to the vehicle-treated group. Based on the volume of fluid milked out from the intestine, 5.45, 54.54, and 61.82% inhibition were recorded for the 150, 300, and 450 mg/kg doses of MEMS, respectively. Based on fluid weight, the 300 and 450 mg/kg doses of the extract showed a significant fluid reduction.

Based on the weight difference of the intestine before and after milking out, 3.1, 47.7, and 56.9% inhibition of fluid were recorded for the 150, 300, 450 mg/kg doses of MEMS, while 61.5% was recorded for the loperamide treated group. However, the extract did not show a significant difference in terms of stool water content from the net weight difference between wet and 48 h dried stool.

### 3.5. *In Vivo* Antidiarrheal Index

The *in vivo* antidiarrheal indices of the extract doses were 34.35, 56.57, and 57.15% for respective oral doses of 150, 300, and 400 mg/kg.

**Figure 1:**
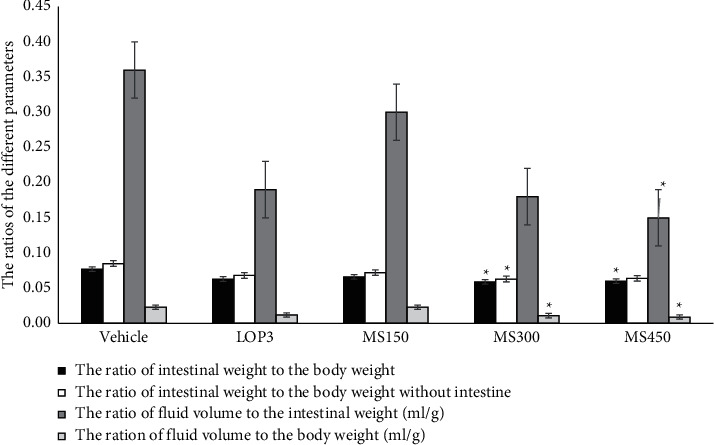
The effect of 80% methanolic leaf extract of *M. stenopetala* on different ratios as a measure of antisecretory or antienteropooling effect. ^*∗*^*p* < 0.05 compared to the negative control, Vehicle = 2% tween 80, LOP3 = loperamide 3 mg/kg, MS150 = 150 mg/kg of *M. stenopetala* extract, MS300 = 300 mg/kg of *M. stenopetala* extract, and MS450 = 450 mg/kg of *M. stenopetala* extract.

Furthermore, the 300 and 450 mg/kg doses of MEMS showed a significant antisecretory activity based on the different ratios derived from the bodyweight of the animal with and without the small intestine and fluid volume milked out (Figure 1). Based on the same ratio parameters, the 450 mg/kg dose demonstrated a significant antisecretory effect, except for the results of the ratio of the weight of the small intestine to the bodyweight of animals, whereas the 150 mg/kg dose of the extract did not produce a significant antisecretory activity.

## 4. Discussion

Castor oil produces diarrhea due to its active metabolite known as ricinoleic acid, which is liberated by the action of lipase in the upper part of the small intestine [23]. By irritating the intestinal mucosa, this acid causes inflammation and release of prostaglandins [24]. Further, the released prostaglandins stimulate the secretion of water and electrolytes into the small intestine. Prostaglandins also stimulate gastrointestinal motility, epithelial permeability, and edema within the intestinal mucosa, thereby preventing the reabsorption of sodium chloride and water [25, 26]. The literature also indicated that the diarrheagenic effect of castor oil is linked with the induction of cytotoxic effect in the intestinal absorptive cells via sodium-potassium ATPase inhibition [27, 28]. This mechanistic evidence supports our use of castor oil as GI motility and secretion inducer in this study.

In the castor oil-induced diarrheal model, the extract resulted in a delay in the onset of diarrhea and reduced the total number of stools, number of wet stools, and weight of wet stools. This is consistent with a study on a hydroalcoholic extract of an Ethiopian plant [29] that suggested the presence of bioactive secondary metabolites to be responsible for the reported effects. A study on the analgesic and anti-inflammatory activities of *M*. *stenopetala* suggested that the observed effects were due to the inhibition of prostaglandin formation [30]. It was also reported that nonsteroidal anti-inflammatory drugs can inhibit the type of diarrhea induced by castor oil [31]. It is therefore plausible to state that the antidiarrheal action of the investigated plant could be due to antiprostaglandin-like effects similar to that of analgesic and anti-inflammatory effects.

Antidiarrheal agents may also act by reducing gastrointestinal motility, which is tested by the charcoal meal test. In this study, the MEMS significantly suppressed gastrointestinal motility, which is evidenced by the significant difference in the ratio of charcoal peristalsis, mainly at doses of 150 and 300 mg/kg, while the 450 mg/kg dose of MEMS showed a significant antimotility effect based on the length of charcoal peristalsis relative to the control group. This may suggest that the MEMS can alter gastrointestinal peristalsis that indicates antimotility activity. This finding is concordant with related studies on other medicinal plants [8, 32].

Moreover, the 300 and 450 mg/kg doses of MEMS demonstrated antisecretory and antienteropooling effect, which is supported by the statistically significant results from the intestinal weight or fluid volume-based ratio parameters. This finding is also suggestive of MEMS's capacity to either inhibit secretion or enhance the absorption of fluid and electrolytes in the lumen, reversing the secretory effect of castor oil. Our study is in line with other similar studies on other medicinal plants with antisecretory effects, which indicated superior effects specifically at the higher dose levels [33, 34].

The ADI is the overall measure of antidiarrheal effect as it considers the combined effects of different values of test models such as percent inhibitions of GI motility, secretion, and percent delay onset time [33, 35]. More effective antidiarrheal effects are those with higher ADI values [36]. The ADI of our study indicated that the antidiarrheal effects of the extract are in a dose-dependent manner.

The antidiarrheal and antisecretory activities of MEMS could be due to the presence of phytochemicals. Various secondary metabolites have been reported to have antidiarrheal activity through different mechanisms. Preliminary phytochemical screening test showed that, based on other works from the literature as well as results from our lab, leaf extracts of M. stenopetala contain alkaloids, flavonoids, tannins, saponins, steroids, phenols, and terpenoids [3, 4]. For instance, tannins exert their antidiarrheal effect through the precipitation of proteins of the enterocytes and thus impede peristaltic movement [37]. Tannins also inhibit the release of prostaglandins, thereby inhibiting motility and secretion induced by castor oil. Moreover, they lower the net intracellular calcium level in smooth muscles to provoke muscle relaxation [38].

Moreover, alkaloids can normalize the permeability of water and electrolytes in the gut [39], whereas flavonoids are designated to possess antidiarrheal activity due to their ability to inhibit intestinal motility. Also, alkaloids and flavonoids inhibit prostaglandin production through alteration of the cyclooxygenase 1 and 2 (COX-1 and COX-2) and the lipoxygenase (LOX) enzyme activity [30]. In addition, the antidiarrheal activity of flavonoids has been attributed to their ability to inhibit intestinal motility and hydroelectrolytic secretion. These compounds also have antioxidant characteristics, which are acknowledged to be responsible for the inhibitory effects exerted upon several enzymes, including those involved in the arachidonic acid metabolism. Therefore, the antidiarrheal and antisecretory activity of *M. stenopetala* leaf crude extract observed in this study could be due to the presence of alkaloids, flavonoids, and tannins in the crude extract [40].

## 5. Conclusions

The result of this investigation revealed that the crude extract of *M. stenopetala* demonstrated antidiarrheal activity. It produced an inhibitory effect on castor oil-induced diarrhea, reduced gastrointestinal motility, and inhibited GI secretion, specifically at the 300 and 450 mg/kg doses of the extract.

## Figures and Tables

**Table 1:** The effect of 80% methanol leaf extract of *M. stenopetala* in the castor oil-induced diarrhea model.

Group	Stool frequency	Stool score	The onset of diarrhea (minutes)	% inhibition of frequency of defecation	% delay of onset of diarrhea
Vehicle	16.50 ± 1.43	1.83 ± 0.14	48.17 ± 8.53	—	-
LOP3	4.50 ± 0.85^*∗*^	1.54 ± 0.19	122.17 ± 19.49^*∗*^	72.7	60.5
MS150	8.50 ± 1.12^*∗*^	1.33 ± 0.18	62.33 ± 22.83^*∗*^	48.5	22.7
MS300	6.83 ± 0.65^*∗*^	0.79 ± 0.11^*∗*^	111.60 ± 27.67^*∗*^	58.6	56.8
MS450	6.50 ± 1.52^*∗*^	1.08 ± 0.20^*∗*^	107.17 ± 17.19^*∗*^	60.6	55.1

All values are expressed as mean ± standard error of the mean (*n* = 6);^*∗*^*p* < 0.05 compared to the negative control, Vehicle = 2% tween 80, LOP3 = loperamide 3 mg/kg, MS150 = 150 mg/kg of *M. stenopetala* extract, MS300 = 300 mg/kg of *M. stenopetala* extract, and MS450 = 450 mg/kg of *M. stenopetala* extract.

**Table 2:** The effect of 80% methanolic leaf extract of *M. stenopetala* on charcoal meal intestinal peristalsis.

Groups	Total intestinal length (cm)	Charcoal peristalsis length (cm)	The ratio of charcoal peristalsis	% inhibition of peristalsis
Vehicle	48.75 ± 2.03	48.75 ± 2.03	1 ± 0.00	—
AT-0.1	54.1 ± 2.11	21.08 ± 4.45^*∗*^	0.38 ± 0.07^*∗*^	61.04
MS150	57.27 ± 1.5	34.75 ± 2.16	0.69 ± 0.03^*∗*^	39.32
MS300	49.25 ± 1.56	35.00 ± 4.46	0.71 ± 0.09^*∗*^	28.93
MS450	46.92 ± 1.61	34.08 ± 3.23^*∗*^	0.74 ± 0.09	27.36

Values are expressed as mean ± standard error of the mean and percent proportions. ^*∗*^*p* < 0.05 compared to the negative control, Vehicle = 2% tween 80, AT-0.1 = atropine sulfate 0.1 mg/kg, MS150 = 150 mg/kg of *M. stenopetala* extract, MS300 = 300 mg/kg of *M. stenopetala* extract, and MS450 = 450 mg/kg of *M. stenopetala* extract.

**Table 3:** The effect of 80% methanolic leaf extract of *M. stenopetala* on fluid secretion parameters.

Groups	The volume of fluid milked out (ml)	% inhibition of volume secretion	Fluid content based on WDI^^^ (*g*)	% inhibition of fluid WDI^^^	Stool water content (*g*)^#^	% inhibition of stool fluid content
Vehicle	0.55 ± 0.15	—	0.65 ± 0.07	—	0.49 ± 0.04	—
LOP3	0.27 ± 0.10^*∗*^	50.91	0.25 ± 0.04^*∗*^	61.5	0.17 ± 0.05^*∗*^	65.2
MS150	0.52 ± 0.17	5.45	0.63 ± 0.15	3.1	0.43 ± 0.10	11.5
MS300	0.25 ± 0.10^*∗*^	54.54	0.34 ± 0.10^*∗*^	47.7	0.22 ± 0.09	54.6
MS450	0.21 ± 0.07^*∗*^	61.82	0.28 ± 0.02^*∗*^	56.9	0.34 ± 0.06	30.5

All values are expressed as mean ± standard error of the mean (*n* = 6); ^*∗*^*p* < 0.05 compared to the negative control, Vehicle = 2% tween 80, LOP3 = loperamide 3 mg/kg, MS150 = 150 mg/kg of *M. stenopetala* extract, MS300 = 300 mg/kg of *M. stenopetala* extract, MS450 = 450 mg/kg of *M. stenopetala* extract, and fluid content was calculated: ^based on WDI (weight difference the intestine) before and after milking out fluid and ^#^based on overall weight differences of wet and dry stool.

## Data Availability

All data supporting the findings are adequately included within the paper.
